# White and gray matter fiber pathways in autism spectrum disorder revealed by ex vivo diffusion MR tractography

**DOI:** 10.1002/brb3.483

**Published:** 2016-05-05

**Authors:** Molly Wilkinson, Rongpin Wang, Andre van der Kouwe, Emi Takahashi

**Affiliations:** ^1^ Department of Behavioral NeuroscienceNortheastern UniversityBostonMassachusetts; ^2^ Division of Newborn MedicineDepartment of MedicineBoston Children's HospitalHarvard Medical SchoolBostonMassachusetts; ^3^ Athinoula A. Martinos Center for Biomedical ImagingMassachusetts General HospitalHarvard Medical SchoolCharlestownMassachusetts; ^4^ Fetal‐Neonatal Neuroimaging and Developmental Science CenterBoston Children's HospitalHarvard Medical SchoolBostonMassachusetts

**Keywords:** Brain, callosal pathways, corticopontine pathways, development, ex vivo imaging, high‐angular resolution diffusion imaging, tractography

## Abstract

**Introduction:**

The goal of this project was to study the white and gray matter brain pathways of young children with autism spectrum disorder (ASD) and investigate how ASD brains differ from those of typically developing children of the same age.

**Methods:**

High angular resolution resolution diffusion imaging tractography and diffusion tensor imaging tractography were used to analyze the brains of two 3‐year‐old children with ASD and two age‐matched controls.

**Results:**

In the ASD brains, the callosal and corticopontine pathways were thinner overall and terminal areas in the cortical gray matter were significantly smaller. The ASD brains had more short‐range u‐fibers in the frontal lobe compared to the control brains. Gray matter pathways were found disorganized with less coherency in the ASD brain, specifically the lateral aspects of the middle part of the brain including motor areas, and both medial and lateral surfaces of the anterior frontal brain regions.

**Conclusion:**

These findings show our tractography technique is useful for identifying differences in brain pathways between the ASD and control groups. Given that scanning the brain of 3‐year‐old children with or even without ASD is challenging, postmortem scanning may offer valuable insights into the connectivity in the brain of young children with ASD.

## Introduction

Autism spectrum disorder (ASD) is a complex neurodevelopmental disorder and a diagnosis received by 1 in 68 children in the United States (Autism and Developmental Disabilities Monitoring Network Surveillance Year 2010 Principal Investigators, 2014). The high prevalence of this disorder is matched with an increasing amount of studies focused on autism, yet for many of the studies regarding white and gray matter, the results have yet to reach a consensus. These contradicting results can be attributed to the range of symptoms and severity found on the autism spectrum, different ages studied, and various types of imaging techniques with varying scan/analyses parameters and regions of interest.

Autism spectrum disorder includes autism, Asperger disorder, and pervasive developmental disorder not otherwise specified. Autism makes sensory processing difficult, so individuals think, learn, process, and interact differently than neurotypical people. Autistic tendencies begin to surface within the first few years of life and the average diagnosis is made at 4 years of age (Autism and Developmental Disabilities Monitoring Network Surveillance Year 2010 Principal Investigators, 2014). A diagnosis of autism can result from a range of observed symptoms pertaining to social, emotional, communication, and processing difficulties, especially regarding communication of their own feelings, opinions, and thoughts with others. ASD individuals are unable to appropriately interpret facial expressions and motives for an action and have difficulty maintaining eye contact.

A cause for autism is still unknown, yet there are multiple theories in place involving genetics, such as high risk genes, de novo mutations, and the increased risk if an individual has an autistic sibling. Environmental factors including chemical and disease exposure during prenatal life and complications during the birth process have also been attributed to an increased risk of developing autism (Landrigan 2010; Gardener et al. 2011).

White matter tracts help transmit information effectively and efficiently, if normally developed, throughout the brain. The corpus callosum is involved in the processing and integrating of higher order information and connecting the two hemispheres of the brain, helping to facilitate interhemispheric information transmission. Autism studies using MRIs to see the corpus callosal pathways have presented consistent evidence of abnormalities in this white matter tract (Egaas et al. 1995; Manes et al. 1999; Hardan et al. 2000). Previous studies found a decrease in size (Lo et al. 2011) and decreases in the number of connections in corpus callosal pathways (Lo et al. 2011; Aoki et al. 2013).

Previous postmortem studies on autism have focused on quantifying the number and size of many components of the brain. Abnormal neuronal migration has been found in histologic studies of ASD brains, as well as irregular neurogenesis in multiple regions (Wegiel et al. 2010). ASD brains between the ages of 4 and 60 years old were used to hypothesize that the varying symptoms and severity of ASD may be a result of different brain regions being affected to varying degrees (Wegiel et al. 2010). Different growth patterns in different areas of the brain lead to different expression levels of the genes associated with those areas. Along with abnormal neurogenesis, neuronal immaturity was spotted in many places. Further research by Wegiel et al. (2014) has focused on the developmental growth patterns in ASD patients and how these patterns may differ from controls. A large decrease in neuron volume, compared to controls, was found in young ASD children. It was found that with increases in age, the closer the brain is to normalizing to a typically developed brain, the lesser alteration in neuronal volume and number is observed. This abnormal growth trajectory may reflect an extreme delay in neuronal growth or severe deficits during early childhood followed by an increase in growth over adulthood.

Postmortem studies of different areas and structures of the brain have revealed that in some regions, the number or density of neurons has increased, while in other regions it may have decreased. This varying pattern of increased and decreased neuronal properties is related to the varying symptoms of the disorder. Histopathologic studies on ASD found a large increase in neuron size and number of neurons within the prefrontal cortex, which is a possible cause for the increased size of the frontal cortex overall (Courchesne et al. 2011). Structural MRI studies also supported this notion, showing the largest overgrowth in gray matter (Palmen et al. 2005; Hazlett et al. 2006). In addition, Casanova et al. (Casanova 2005) found that an altered minicolumn structure in ASD brains seems to play a role in autistic symptoms, especially seizure activity, and differences in white and gray matter in autism.

Diffusion tensor MR imaging (DTI) has been used in many studies of white matter. DTI is a noninvasive MRI technique that traditionally has been used for the mapping of white matter tracts throughout the brain by measuring the direction of water diffusivity. A significant decrease in the volume and number of streamlines within the anterior region of the corpus callosum was found in a DTI study of 11 children and adolescents with ASD (Schaer et al. 2013). Decreases in local Gyrification Index within the right inferior frontal region reaching into the inferior parietal lobe and in the right medial parieto‐occipital region were found as well. Decreases in gyrification and long‐range connectivity have been measured in many other ASD studies, and Schaer and colleagues have produced similar results (Schaer et al. 2013). Fractional anisotropy (FA) was found to be decreased in several areas of the right hemisphere in ASD; which is also consistent with the results of other studies which have mostly found decreased FA for this age group. An interesting result from Schaer et al. is that FA reductions could only be found in the right hemisphere. A relationship between the local Gyrification Index and short‐range connectivity was established through this study. The ASD patients showed a higher value for the local Gyrification Index correlated with a higher degree of short‐range connectivity. Schaer et al. hypothesized that this relation may stem from the autistic brain's desire to compensate for its differing patterns of tract connectivity during younger developmental stages. Other DTI studies have shown decreased structural connectivity between the frontal cortex and other regions, such as the putamen and nucleus accumbens (Langen et al. 2012).

A unique study used autistic subjects and controls between the ages of 8 and 18 years old as the means to investigate the differences between DTI and High angular resolution resolution diffusion imaging (HARDI) techniques (Eavani et al. 2011). Identification of the arcuate fasciculus was used to evaluate the two imaging techniques. The length and FA of the arcuate fasciculus of the ASD and control subjects showed significantly decreased values in the DTI results when compared to the HARDI results of both ASD and control arcuate fasciculi. The inability of DTI to fully detect the arcuate fasciculus may also extend to other tracts of the brain and serves as a disadvantage.

Our study takes a closer look at the brains of ASD patients at a very young age through DTI and HARDI, shortly after and during the time of many key developmental changes. Looking at young ages is important to establish triggers of ASD atypical development. In this study, a higher number of short‐range u‐fibers were seen in ASD patients. Functional MRI studies have shown that the frontal lobe is the only region within the anterior part of the human ASD brain that shows local hyper‐connectivity (Keown et al. 2013), and increases in functional connectivity within frontal cortex structures (anterior cingulate cortex, middle frontal gyrus, paracingulate gyrus, and orbitofrontal cortex) are correlated with ASD symptom severity. However, whether DTI can also detect such local, fine connectivity in ASD individuals is still unknown.

HARDI tractography is another type tractography is another type of diffusion imaging technique including postprocessing data analyses that may be preferable over DTI, as HARDI is able to distinguish crossing fibers within a voxel even in immature brains (Takahashi et al. 2012), which are typically more challenging to segment due to a surplus of unmyelinated fibers. Both HARDI and DTI were used in this study. The way we prepare the specimens (Takahashi et al. 2010), high performance MR gradients, and custom‐made coils together allow imaging of high‐resolution fiber pathways in ex vivo brains.

The goal of this project was to study the white and gray matter in the brains of young children with ASD and investigate how it differs from the white and gray matter of developmentally typical children of the same age. HARDI tractography and DTI were used to analyze the brains of two 3 year old children with ASD and two controls. We hypothesized that the ASD brains would present with increased short‐range connectivity compared to controls.

## Methods

### Specimens

The research was conducted in accordance with the Helsinki Declaration, and approved by the Boston Children's Hospital Human Research Committee. Brain specimens of two children with ASD and two children with no neurological/pathological findings were received from the University of Maryland Brain and Tissue Bank, under a material transfer agreement between the Brain Bank and the Boston Children's Hospital. Cases 1 and 3 are diagnosed with autism, and Cases 2 and 4 are controls. All of the specimens were from the right hemisphere.

Case 1, M4021M, is the tissue of a 3 years and 114 days old African American male, diagnosed with autism, who died from a drowning accident. The family history report included a mentally ill maternal cousin, a brother with a speech delay, and a father that stutters. The neurology report stated a grossly normal brain and the autopsy reported pulmonary edema and congestion, as well as cerebral edema. The postmortem interval was 15 h and as of March 2015 this brain has been in storage for 12 years and 174 days.

Case 2, #5282, is the control for Case 1, and is the brain tissue of a 2 years and 305 days old Hispanic male. Prior to death from choking, the child was reported healthy and was not on any medications. Cause of death (choking) would have no detrimental effect on the white matter pathways within the brain nor the gray matter examined in this study. No previous neurological conditions were found, therefore the brain is an appropriate subject for a control in this case. The postmortem interval was 16 h and as of March 2015 this brain has been in storage for 6 years and 334 days.

Case 3, M4029M, is the tissue of a 3 years and 274 days old African American male with autism and delayed speech, who died from a drowning incident. Neurology report included anoxic‐ischemic encephalopathy in the basal ganglia and acute brain edema. The autopsy found pulmonary edema and congestion, as well as cerebral edema. The postmortem interval was 13 h and as of March 2015 this brain has been in storage for 12 years and 143 days.

Case 4, #5608, the control for Case 3, is a 3 years and 194 days old Caucasian male who was born with a ventricular septal defect. Prior to death, the child had three open heart operations and intestinal surgery. Neurology reported a small brain for his age, but otherwise grossly normal. Cause of death (drowning) would have no detrimental effect on the white matter pathways within the brain nor the gray matter examined in this study. No previous neurological conditions were found; therefore the brain is an appropriate subject for a control in this case. The postmortem interval was 29 h and as of March 2015 this brain has been in storage for 3 years and 74 days.

**Figure 1:**
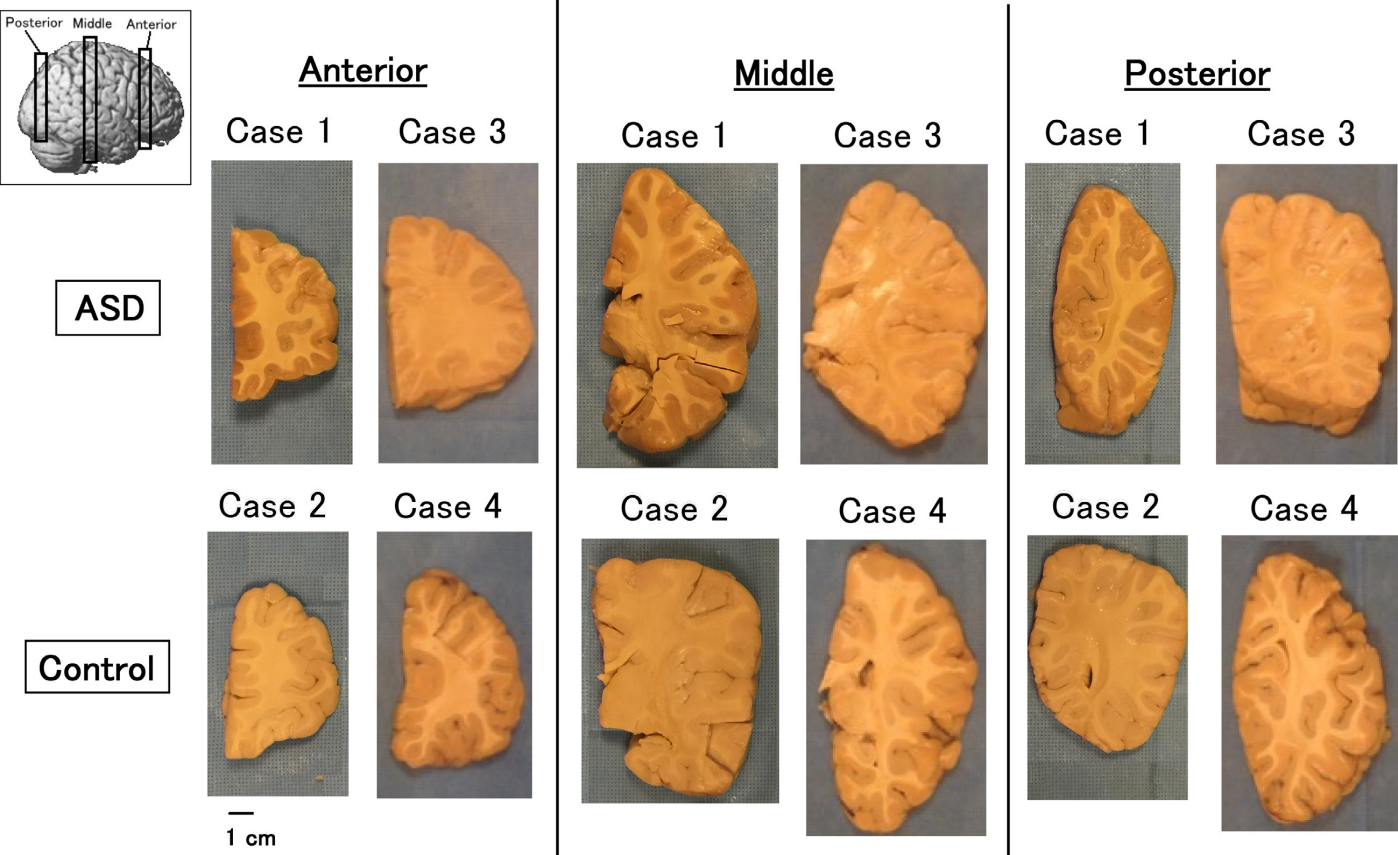
Brain specimens studied. See details in text. Approximate slice locations are shown in left upper corner (three‐dimensional brain image is from http://www.fil.ion.ucl.ac.uk/spm).

Three coronal brain slices from anterior, middle, and posterior brain regions were obtained for each ASD and control brain (See Fig. 1 left upper corner for approximate slice locations in the whole brain). The anterior slice was from a slightly anterior part of the anterior edge of the lateral ventricles, and included the superior, middle, and inferior frontal gyri in the prefrontal cortex as well as the orbitofrontal cortex on the bottom. This section approximately corresponded to Brodmann's Areas (BAs) 8 (orbitofrontal cortex), 9, 46, 45 (pars triangularis), 47 (inferior prefrontal cortex), and 11 (orbitofrontal cortex) in the lateral surface in this order, and areas 8, 32, and 12 in the medial surface in this order. The middle slice included the pre‐ and postcentral gyri, insula, and superior, middle, and inferior temporal gyri. This section approximately corresponded to BAs 4/6 (primary/supplementary motor areas), 3/1/2 (primary somatosensory areas), 41/42 (primary auditory areas), and 22/21/20 in the lateral surface in this order. The striatum was located subcortically. The posterior slice is slightly posterior to the posterior edge of the lateral ventricles, and included angular and lateral occipital gyri (BAs 39 and 19) in the lateral surface.

**Figure 2:**
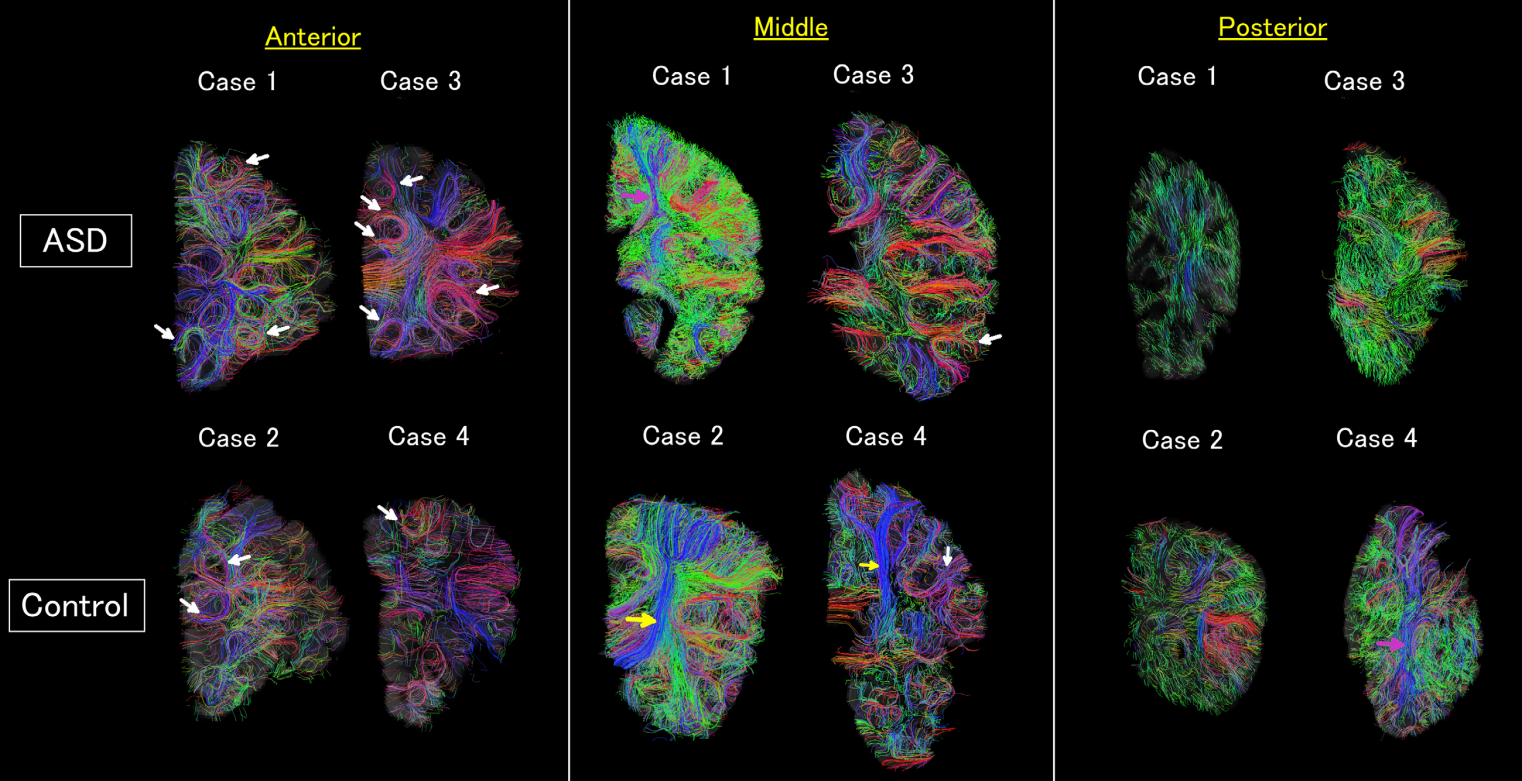
Diffusion tensor imaging (DTI) tractograpy. The color‐coding of tractography pathways was based on a standard red‐green‐blue (RGB) code that was applied to the vector in each brain area to show the spatial locations of terminal regions of each pathway (red for right‐left, blue for dorsal‐ventral, and green for anterior‐posterior). White arrows: example short‐range u‐fibers, yellow arrows: long‐range pathways that were more coherent in DTI tractography compared to high angular resolution diffusion imaging (HARDI) tractography, pink arrows: long‐range pathways that were identified with a similar coherency in both DTI and HARDI tractography.

**Figure 3:**
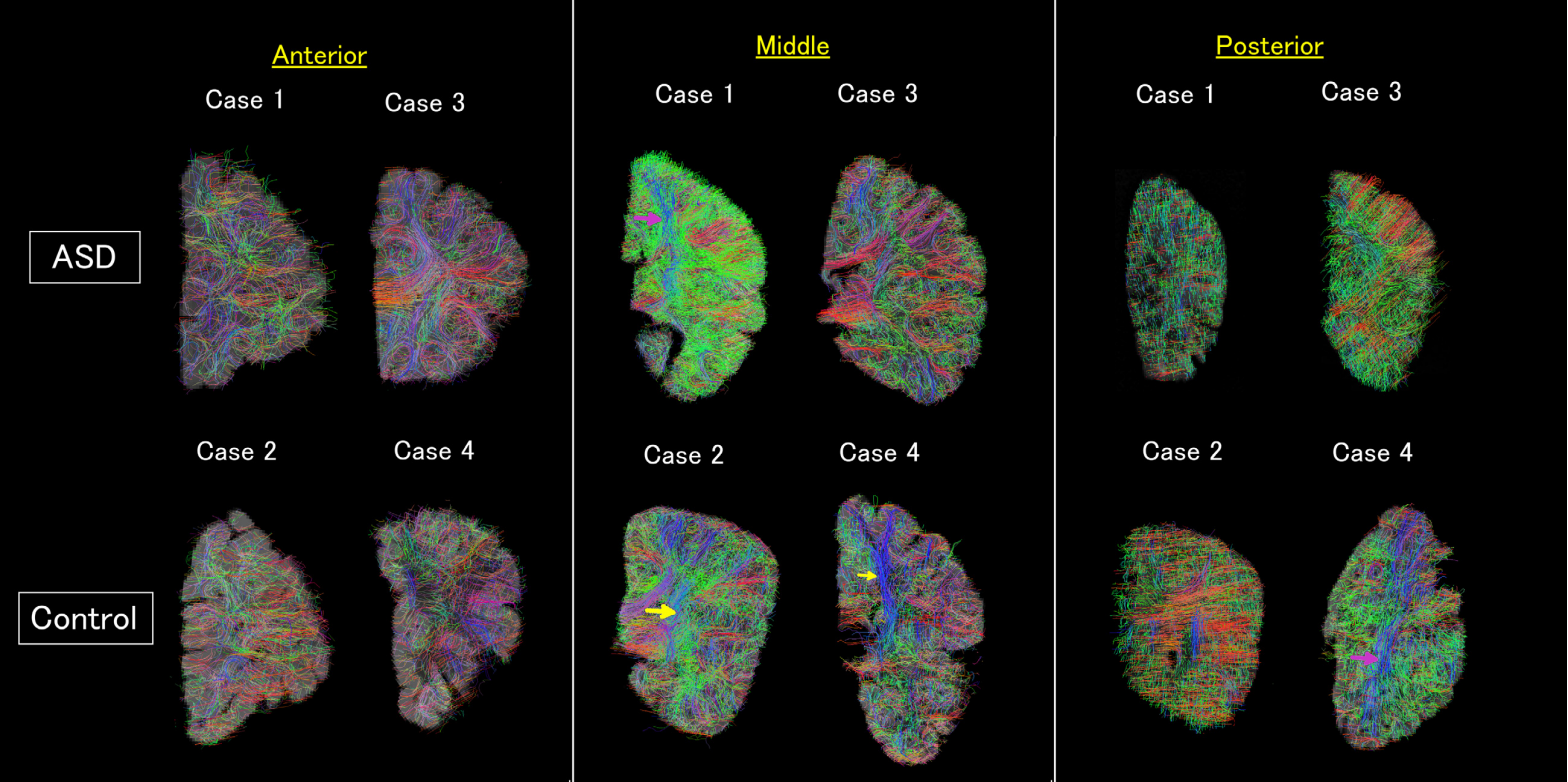
High angular resolution diffusion imaging (HARDI) tractography. The color‐coding of tractography pathways was the one used in Figure 2. Yellow arrows: long‐range pathways that were more coherent in diffusion tensor imaging (DTI) tractography compared to HARDI tractography, pink arrows: long‐range pathways that were identified with a similar coherency in both DTI and HARDI tractography.

The slices were approximately 1 cm thickness, except for the middle slices of Cases 1 and 3, with approximately 2 cm thickness. This thickness difference affected the apparent looking of fiber pathways in Figures 2, 3: using the same analyses and filter conditions specifically used in this study, thicker slices with more imaged voxel show more tractography pathways because we initiate tracking from each imaging voxel. After the MRI scan, these slices were sent back to the University of Maryland Brain and Tissue Bank.

To confirm the courses and destinations of long‐range tractography pathways, we also used a whole adult brain specimen obtained from the Harvard Medical School with no neurological and pathological reports.

### Preparation for MRI scan

The slices were prepared for the MRI scans by being placed into individual ziploc bags with Fomblin oil (same as e.g. Takahashi et al., 2010), and then placed parallel to each other in a box with plastic boards placed in between the slices. The scans were done at the Athinoula A. Martinos Center for Biomedical Imaging.

### MRI scan parameters

Diffusion‐weighted data were acquired over two averages using a steady‐state free‐precession (SSFP)‐based diffusion sequence (trufi) (TR/TE = 24.82/18.76 msec, *α *= 60°) (McNab and Miller, 2008), using a 3 Tesla Siemens TIM Trio MRI machine with a 32 channel head coil, with a bandwidth 100 Hz/px. Imaging matrix was 200 × 400 × 200 for the slice specimens, and 176 × 128 × 192 for the whole brain. Slab thickness 192 mm with 240 slices (800 *μ*m thick slices), 200 × 200 matrix with 160 mm square Field of View (FOV) (800 × 800 *μ*m in‐plane resolution). Diffusion weighting was performed along 44 directions (*b* = 800 sec/mm^2^) with 4 *b* = 0 images. The diffusion directions were generated by electrostatic repulsion on the surface of a sphere to ensure approximately equidistant spacing. Total scan time was 17 h 35 min 24 sec.

With an SSFP diffusion scan, achieving a much higher *b* value is difficult, given the long scans and constraints on gradient heating. The use of SSFP is relatively new, which was necessary to achieve high‐resolution ex vivo diffusion.

### Reconstruction and identification of tractography pathways

White and gray matter tracts were reconstructed using Diffusion Toolkit, and visualized in TrackVis software (http://trackvis.org). A streamline algorithm for diffusion tractography was used (Mori et al. 1999), as in previous publications (Takahashi et al. 2010, 2012). The term “streamline” refers to connecting tractography pathways using a local maximum (of tensors in DTI) or maxima (of orientation distribution function [ODF] in HARDI). This method is true for both DTI and HARDI. We used all the local maxima of ODF to produce HARDI tractography pathways.

Trajectories were propagated by consistently pursuing the orientation vector of least curvature. Tracking was terminated when the angle between 2 consecutive orientation vectors was greater than the given threshold (60°) or when the fibers extended outside of the brain surface. For the latter determination, brain mask images created by Diffusion Toolkit were used in order to determine coherence within the brain and not in the surrounding immersion fluid. Brain mask volumes were used to terminate tractography structures instead of the FA threshold because progressive myelination and crossing fibers in the developing brain can result in low FA values that may potentially incorrectly terminate tractography tracing in the gray matter (Wedeen et al. 2005; Takahashi et al. 2010, 2012). Although we have been using this strategy in our past studies, the use of an FA threshold when performing diffusion tractography is still a common tractography method, so our approach is unique in that sense. Since there are relatively large low FA regions in immature white matter, it is important to consider not using an FA threshold for tractography in infants and children. This issue may also apply to tractography in adults as FA values tend to decrease potentially to lower values than a given threshold towards the edge of white matter.

For visualization purposes in Figures 2, 3, we restricted the percentages of the number of detected tractography connections by the demonstration of 5% of the total detected connections that touched a coronal slice with a thickness of 1 voxel (80 *μ*m). In Fig. 5, we used a coronal slice filter with a thickness of 5 voxels (4 mm) and restricted to show only pathways both of the terminal points of which are within the slice filter in order to see short‐range gray matter pathways. We showed 30% of the total detected pathways in Fig. 5.

Overall distribution of u‐fiber pathways were first investigated by eye (Fig. 2). We then quantified the number of u‐fiber tracts and track count, volume, and length of each u‐fiber tract (Table 1) by placing a region of interest (ROI) in each gyrus and by restricting pathways using another ROI in adjacent gyral structures. No length threshold was used.

**Table 1:** U‐fibers in autism specimen

Specimen	Track count	Volume (ml)	Length (mm)
Anterior			
M4021M	416	0.68	29.49 ± 10.55
	365	0.46	18.30 ± 6.12
	261	0.48	22.86 ± 9.79
	149	0.22	15.42 ± 7.61
	113	0.29	19.82 ± 9.52
	96	0.35	27.11 ± 11.71
	52	0.31	35.34 ± 13.49
	30	0.14	25.83 ± 4.81
	18	0.08	28.13 ± 1.65
M4029M	452	0.73	21.81 ± 12.51
	371	0.88	70.17 ± 54.79
	149	0.51	40.01 ± 15.06
	125	0.29	30.54 ± 5.74
	102	0.12	17.16 ± 1.61
	94	0.31	28.45 ± 13.52
	83	0.23	18.19 ± 4.38
	63	0.15	11.59 ± 6.96
	55	0.21	32.28 ± 13.26
	41	0.15	22.91 ± 13.48
	41	0.13	27.41 ± 3.44
	17	0.14	35.51 ± 17.14
Middle			
M4021M	587	0.89	25.29 ± 12.00
	280	0.63	29.27 ± 5.57
	42	0.11	17.36 ± 2.87
M4029M	777	0.97	27.52 ± 10.32
	103	0.37	28.69 ± 11.77
	92	0.40	38.61 ± 11.41
	68	0.40	45.68 ± 11.94

**Figure 4:**
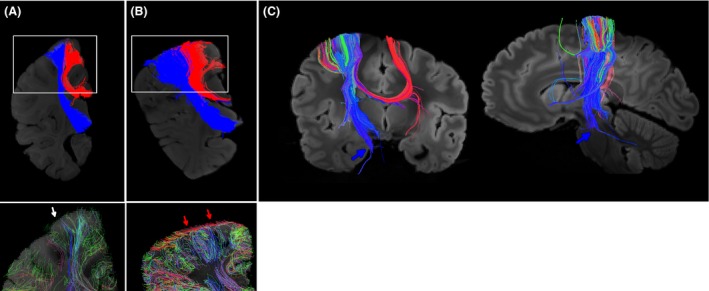
Callosal and corticopontine pathways and gray matter pathways in regions where those pathways terminate. (A, B, upper panels) diffusion tensor imaging (DTI) tractography on Case 1 (A) and Case 2 (B). Callosal pathways are shown in red, and corticopontine pathways are shown in blue. Regions indicated by white rectangles are magnified in lower panels. (A, B, lower panels) high angular resolution diffusion imaging (HARDI) tractography on Case 1 (A) and Case 2 (B). Gray matter pathways are shown in brain regions where the callosal and corticopontine pathways were terminated. Red arrows indicate coherent tangential pathways within the gray matter of the control brain, and white arrow shows that the Autism spectrum disorder brain has only very sparse pathways without clear directionality, under the given tractography condition (see details in the methods section). (C) DTI tractography on a healthy adult brain. The course and destination of the corticopontine pathways (mostly blue pathways) were confirmed. Blue arrows indicate the pontine region where a part of the corticopontine pathways was terminated and a few pathways continue projecting to the cerebellum. The color‐coding of tractography pathways was the one used in Figure 2.

**Figure 5:**
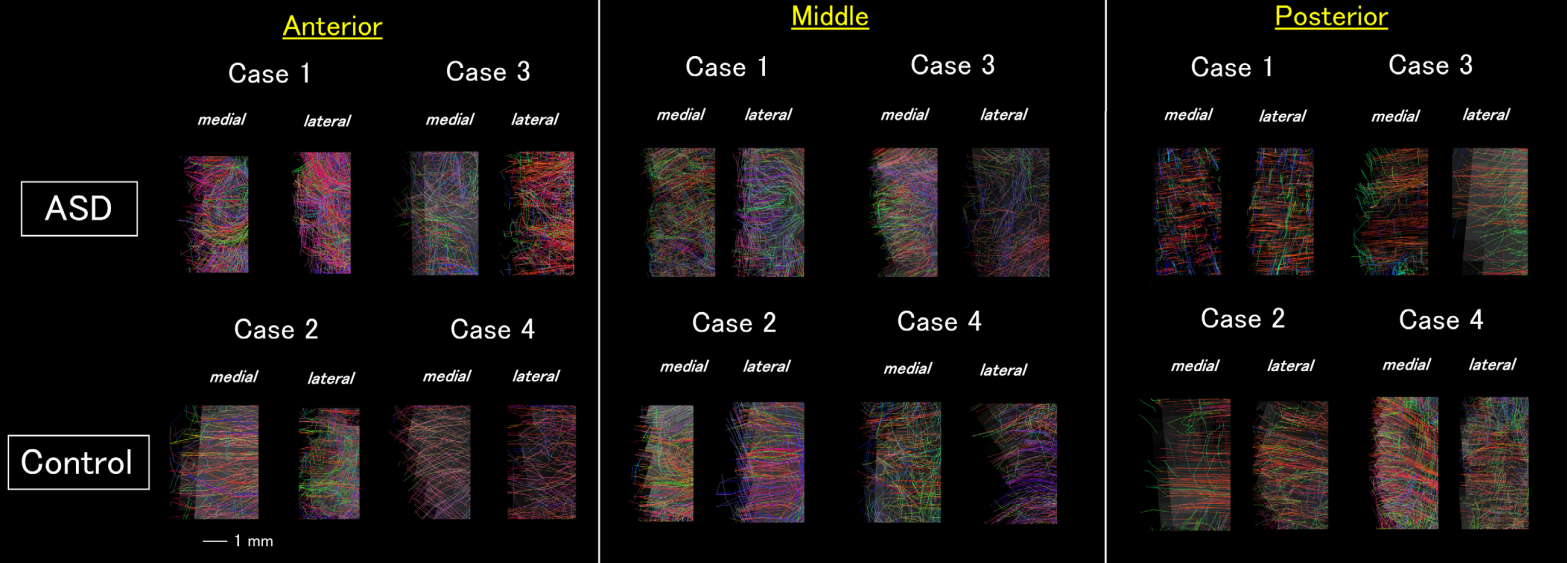
High angular resolution diffusion imaging tractography of the gray matter pathways. Both medial and lateral surfaces of the anterior slices showed clear disorganization in the Autism spectrum disorder brains. In the medial slices, the lateral surface showed more sparse, less coherent pathways compared to the controls. The posterior slices did not show clear difference between two groups. The color‐coding of tractography pathways was the one used in Figures 2 and 3.

The color‐coding of tractography pathways in Figures 2, 3, 5, and panels in Figure 4, except for the two panels for the callosal and corticopontine pathways in ASD and normal specimens, was based on a standard red‐green‐blue (RGB) code that was applied to the vector in each brain area to show the spatial locations of terminal regions of each pathway (red for right‐left, blue for dorsal‐ventral, and green for anterior‐posterior). In the two panels for the callosal and corticopontine pathways in ASD and normal specimens, we used red for the callosal and blue for the corticopontine pathways for the visualization purpose.

## Results

The size of the brain and the volume of the white matter and gray matter were not clearly different by eyes in the three coronal slice regions of the brain (Fig. 1).

### White matter pathways

Diffusion tensor imaging and HARDI tractography showed similar structural coherency in many brain regions. However, we noticed that DTI revealed stronger, more coherent white matter pathways compared to HARDI.

For example, the anterior and middle brain slices showed clearer short‐range u‐fibers in both ASD and control brains (Fig. 2, white arrow as examples of u‐fibers). We quantified track count, volume, and length of the detected u‐fibers in brains with ASD (Table 1) and control brains (Table 2). Although a small sample size was used, the difference between ASD and control brains was clear in that the ASD brains had more u‐fibers in the anterior slices compared to controls. In the middle and posterior slices, long‐range white matter pathways were clearer in some of the brains, and the others showed similar long‐range coherency in those tracts (Figs. 2, 3, yellow arrows for stronger coherency in DTI, and pink arrows for similar results between DTI and HARDI).

**Table 2:** U‐fibers in control specimen

Specimen	Track count	Volume (ml)	Length (mm)
Anterior			
5282	195	0.52	47.60 ± 19.66
	139	0.35	26.03 ± 7.73
	103	0.31	28.77 ± 13.18
	41	0.12	18.99 ± 6.48
	39	0.06	9.99 ± 2.02
5608	56	0.14	20.93 ± 4.44

We identified in the middle brain slices two types of long‐range white matter pathways likely corresponding to the callosal and corticopontine pathways (Fig. 4A,B, upper panels). Both callosal (shown in red) and corticopontine (shown in blue) pathways were thinner (diameter 4 mm [ASD] vs. 8 mm [control] for both callosal and corticopontine pathways at the location where fibers exit from gyri), and the terminal areas in the cortical gray matter were smaller (lengths of the cortical surface occupied by callosal: 2 mm [ASD] vs. 3.2 mm [control], corticopontine: 1.6 mm [ASD] vs. 3.0 mm [control] in the slices shown in Fig. 4) in the ASD brain compared to the control brain. For the corticopontine pathways, we confirmed the course and the destination of the tracts using ex vivo whole brain tractography data obtained from a normal adult (Fig. 4C).

### Gray matter pathways

We first investigated the gray matter in the terminal regions of the corpus callosal and corticopontine pathways described above (Fig. 4A,B, lower panels). Using a coronal slice filter described in the method section, clear differences were observed in the gray matter between the ASD and control brains. The control brain showed strong tangential coherency (red arrows; red pathways close to the surface), while the ASD brain showed very sparse pathways without clear directionality (white arrow).

Next, we examined gray matter pathways in the medial and lateral brain surfaces in the scanned slices (Fig. 5). The medial surface regions shown in Figure 5 were located in the cingulate gyrus, and the lateral surface regions were located around the middle part of the lateral surface (middle/inferior frontal regions (around BAs 44 and 45 [Broca's area] in the anterior slice, around BAs 41 and 42 [primary auditory area] in the middle slice, and around BA 19 in the posterior slice). The coherency of gray matter pathways was overall less in the ASD brains but with regional variability. Posterior slices did not show a clear difference in gray matter pathways between the ASD and control brains, while lateral surface of the middle slices and both medial and lateral surfaces of the anterior slices showed abnormal, multi‐directional patterns of gray matter coherency in the ASD brains (Fig. 5).

## Discussion

We used high‐resolution diffusion MR imaging tractography on brains with ASD and typically developing brains ex vivo. Corpus callosal and corticopontine pathways were thinner and terminal regions in the cerebral cortex were narrower in the ASD brain. Gray matter pathways were found disorganized with less coherency in the ASD brain, specifically the lateral aspects of the middle part of the brain including motor areas, and both medial and lateral surfaces of the anterior frontal brain regions. U‐fibers existed in a greater amount in the frontal lobe of the ASD brains. The terminal regions of the callosal and corticopontine pathways in the cerebral cortex also showed abnormal gray matter pathways within the cortex of the ASD brain. These results suggest that our tractography technique is useful for imaging detailed brain pathways, especially in the gray matter, and has potential to apply to wider brain regions of ASD in the near future. The HARDI tractography offers imaging with high resolution, and using HARDI to study fiber trackings in post‐mortem brains will probably offer an even higher resolution due to the possibility of scanning the brain for longer times, and due to no motion or pulsative artifacts. Scanning the brain of 3‐year‐old children is challenging, and doing it in such young children with ASD is likely even more challenging. Therefore, post‐mortem scanning may offer valuable insights into the connectivity in the brain of young children with ASD, which together with existing knowledge from fiber tracking and brain activity in clinical samples of ASD can contribute to new understandings of the pathogenesis of ASD.

### White matter abnormalities – callosal pathways

Results for the callosal pathways in ASD brains are consistent with findings from previous tractography studies. The callosal pathways in the middle sections were found to be thinner, with smaller terminal areas in ASD brains when compared to controls in this study. Previous studies have presented with similar results. A study comparing ASD children, developmentally delayed children, and typically developing controls between the ages of 3 and 4 years old found that ASD children have a smaller corpus callosum than controls of the same age (Boger‐Megiddo et al. 2006). This was examined through an investigation of the mid‐sagittal slice of the corpus callosum. The corpus callosum volume was adjusted in ASD children accordingly due to their increase in cerebral cortex volume. With appropriate scaling, the corpus callosum in ASD was found to be smaller and the volume of the corpus callosum did not proportionately increase with overall cerebral cortex volume. Decrease in volume and size of the corpus callosum has been replicated in other MRI and imaging technology studies, as well (Thomas et al. 2011; Prigge et al. 2013; Hanaie et al. 2014). The reduced volume of the corpus callosum as a whole, not regionally, in ASD has also been found in autistic adolescents and adults (Egaas et al. 1995; Gardener et al. 2011). A decrease in the volume or the thickness of the callosal tracts results in decreased interhemispheric and long‐range connectivity of the corpus callosum.

### Potential uses of our technique to the study using fractional anisotropy

Decreased connectivity may also occur through a decrease in myelination, which can be represented by a decrease in FA. Other studies of ASD individuals have shown a reduced FA value when compared to controls, in the corpus callosum (Kumar et al. 2010), as well as other pathways (Ellmore et al. 2013). Travers et al. (2015) performed a longitudinal DTI study of the corpus callosum in 100 ASD males over 9 years. Their results included an increase of FA values in ASD during the first couple years of life when compared to controls, then followed by a period of FA decrease during childhood in the ASD participants and an increase in developmentally typical participants. Overall, the ASD group provided evidence for a decrease in FA of the corpus callosum. Many findings of reductions in corpus callosum FA values were found in autistic adolescents and adults (Shukla et al. 2011). In the current study, we did not quantify FA values having only two brains in each group of the ASD and control. However, our tractography method has a great potential for the study of FA values as discussed below.

Important differences in their study compared to our current and previous studies are not only the difference between DTI and HARDI, but more importantly their use of an FA threshold for tracking pathways. As we explained in the method section, we do not use an FA threshold in our studies in order to identify immature pathways. By using such a threshold, it is possible to miss a whole picture of the abnormalities of brain pathways. For example, we identified in this study accurate gray matter regions (low FA areas) where callosal pathways were terminated, and found abnormal pathways within the gray matter. Furthermore, white matter pathways in ASD individuals may include pathways with further lower FA values than the given FA threshold, which is potentially important to be assessed in order to see accurate pathology of the pathways. It would also be useful to resolve controversies of FA values in ASD patients.

### White matter abnormalities – short‐range u‐fiber pathways

We observed that ASD brains had more short‐range u‐fibers, especially in the frontal lobe compared to the control brains. This finding is consistent with previous functional MR connectivity studies that proposed local over‐connectivity in the ASD frontal lobe (Keown et al. 2013), and we showed that diffusion tractography can support their notion.

### White matter abnormalities – corticopontine pathways

The corticopontine pathways in the human brain are known to be involved in saccadic eye movement. Previous studies report abnormalities in saccadic eye movements in individuals with autism, being most severe during childhood and decreasing with age (Johnson et al. 2012; Schmitt et al. 2014). In our study, the corticopontine pathways were thinner and the areas of termination where the pathways ended were smaller than the corticopontine pathways in the control brains. These results are consistent with the literature, showing the corticopontine pathways are abnormally underdeveloped in young children with autism.

### Gray matter abnormalities

Interestingly, we found abnormal pathways within the gray matter in the ASD brain where the callosal and corticopontine pathways terminated. The gray matter abnormalities that were found in this comparison between 3‐year‐old ASD and developmentally typical brain tissue were that the medial and lateral surfaces of the anterior slices and the lateral surface of the middle slices of the ASD brains showed multi‐directional, disorganized gray matter. Slices of the control brains showed uniform, unidirectional gray matter patterns. Our high‐resolution diffusion MR tractography technique has been useful for the study of gray matter pathways in developing human population starting from fetal ages. Our tractography technique successfully imaged the multi‐directional, disorganized pattern that has been noted previously in histopathologic studies on ASD, as well as overall abnormalities of gray matter in autistic individuals. The detected abnormal disorganization of gray matter tractography pathways in ASD provides evidence that this is the first study showing fine gray matter pathways in ASD using MR tractography. In our study, the lateral surface of the middle slice and the medial and lateral surfaces of the anterior slice of the ASD brains showed abnormalities in gray matter organization.

### Role of white matter abnormalities in autism symptoms

White matter is essential for correct information transmission throughout the brain within a short amount of time. Without typical myelination development, signals can be transmitted to the wrong areas of the brain, they may never get sent anywhere at all, or they can arrive after a long period of time, which can sometimes cause devastating effects. Many human‐specific capabilities are affected in autistic individuals, causing functional difficulties and delays with delayed memory, executive functioning, immediate memory, language, speed of thought, visuospatial processing, and working memory. Vasquez and Zakzanis (2015) performed a study focused on white matter defects and found that in ASD patients all of the above symptoms were related to white matter abnormalities. Our study presents a decrease in the tract length and volume of the ASD corpus callosum, a major white matter tract in the human brain, providing further evidence that white matter deficits occurring during early developmental stages are one of the main causes or effects of autism. Finding a cause, or many causes, to autism will help to target treatment and therapies to that specific region of the brain or functional system within the body. Future research should continue to focus on the altered white matter found throughout the autistic brain in order to build a highly focused and personalized approach to the treatment of autism. Closer looks at when during the developmental process autistic patients tend to stray from the typical developmental course of white matter will assist in early intervention methods for future autistic individuals and help to decrease, or eliminate, the severity of the disorder for patients.

### Limitation of the current study and future directions

Although our tractography technique is innovative to study local, gray matter pathways, one limitation of the current study is due to the use of sliced brain specimens. Unfortunately, pathways running in the anterior‐posterior direction could not be clearly identified in the coronal slices; the callosal and corticopontine pathways were the only two pathways clearly identified in these slices. Another limitation that should be noted is that brain edema is not reported in the control subjects, cases 2 and 4, but was mentioned in the pathology reports of the autistic patients, cases 1 and 3. Although drowning does not affect white matter pathways, it has been previously documented that edema causes changes in DTI measures.

Over many decades, there have been hypotheses and mounting evidence linking fiber maturation and gyral formation. Typical gyrification is representative of developmentally normal cortical expansion throughout the human brain. There has been growing awareness that developmental disturbances of gyral folding can lead to neurological disorders, including autism (Levitt et al. 2003), and a number of studies have shown abnormal white matter development in such disorders, suggesting altered brain connectivity. A number of theories regarding the link between developing white and gray matter connectivity and cortical folding are still actively debated. Our technique has an advantage to study gray matter connectivity, and therefore it is promising to study the relationships between gyrification and gray matter fiber pathways using our technique.

While HARDI has theoretically better concepts, we are aware that DTI sometimes outperform HARDI under specific conditions. With HARDI, crossing pathways could be better differentiated, but continuous trajectories curving with angles above the given angle threshold are hard to detect, while DTI works to continue such pathways. Given the small sample size of the current study, it is hard to conclude/exclude the possibility that that there are individual variabilities regarding which algorithm works better to identify more accurate pathways. However, our results suggest that it is important not to exclude the potential of a conventional tensor fit in the future study.

## Conflict of Interest

None declared.
